# Sensing of the Microbial Neighborhood by *Candida albicans*

**DOI:** 10.1371/journal.ppat.1003661

**Published:** 2013-10-31

**Authors:** Emily M. Mallick, Richard J. Bennett

**Affiliations:** Department of Molecular Microbiology and Immunology, Brown University, Providence, Rhode Island, United States of America; Duke University Medical Center, United States of America

## Dual Identities: *Candida albicans* as Human Commensal and Opportunistic Pathogen

*Candida albicans* is a polymorphic fungus that inhabits a variety of niches in healthy human bodies. In addition to being a component of the normal microbiota, *C. albicans* is an opportunistic pathogen that causes superficial mucosal infections as well as disseminated disease. Importantly, *C. albicans* that is part of the normal microbiota is responsible for seeding these infections [1]. As the fourth most common cause of nosocomial infections, *C. albicans* is commonly isolated from immunocompromised individuals, including those with HIV, those immunosuppressed due to cancer treatment, and premature babies [2]. The ability of this fungus to present as both as a commensal and as a life-threatening pathogen is due, in large part, to its ability to sense and react to the environment. *C. albicans* uses quorum sensing to react to other *Candida* cells, pheromone signaling in the context of mating and sexual biofilm formation, and a variety of mechanisms for interkingdom interactions with the bacterial microbiota. This article highlights the ways in which *C. albicans* cells signal both to one another and to other microbial species.

## Quorum Sensing in *C. albicans*

**Figure 1:**
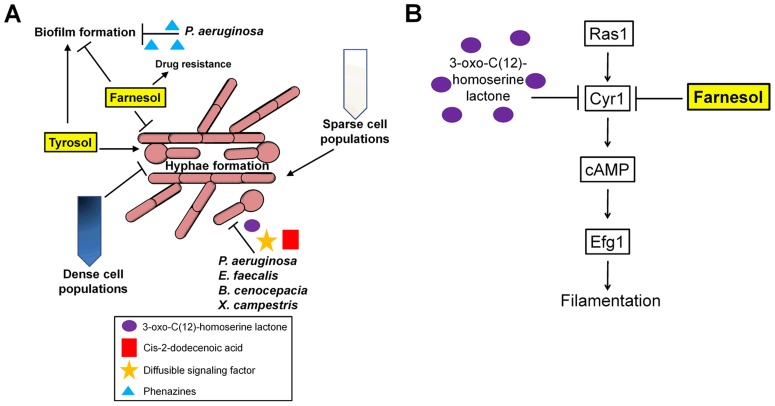
Environmental cues sensed by *C. albicans*. (**A**) Schematic representation of how *C. albicans* morphology and biofilm formation is regulated by quorum sensing and signaling with other microbial species. (**B**) Farnesol and 3-oxo-C(12)-homoserine lactone both act on the Ras1 pathway to inhibit the yeast-to-hyphal transition by inhibiting Cyr1 and cAMP signaling.

*C. albicans* virulence depends on its ability to switch between distinct morphologic and phenotypic states, and these transitions are directly influenced by its environment. Quorum sensing (QS) is used by *C. albicans* to communicate with other *Candida* cells, and is driven by soluble quorum-sensing molecules or autoinducers that are secreted into the environment in a density-dependent manner [3], [4]. QS regulates several pathogenic traits including hyphal (filamentous) growth. This phenomenon is evident by the “inoculum effect,” in which the formation of hyphae is repressed in cells grown at high densities, while cells grown at low densities are able to germinate [5], [6] (**Figure 1A**). Several key QS molecules have been identified that have antagonistic effects, including farnesol and tyrosol. Farnesol inhibits the yeast-hyphal transition by inhibiting adenylate cyclase (Cyr1), part of a central regulatory pathway that impacts filamentous growth [5]–[8] (**Figure 1B**). Conversely, tyrosol shortens lag-phase growth in low-density cultures and stimulates germ-tube formation in yeast cells [9]. Other molecules that are potential QS molecules in *C. albicans* include phenylethyl alcohol, tryptophol, and MARS (morphogenic autoregulatory substance), although the mechanisms of action of these molecules remain unclear [10]–[12]. Thus, multiple QS molecules can impact *C. albicans* morphology (**Figure 1**).

Quorum sensing also regulates the formation of biofilms, which are structured communities of yeast cells and hyphae that form on host tissues or the surface of implanted medical devices. These structures also accrue an extracellular matrix that is made up of carbohydrates including β-1,3 glucan [13]. As the QS molecule farnesol inhibits filamentation, it also acts to suppress overall biofilm formation [14]. However, farnesol and possibly other filamentation-repressing QS molecules may also promote biofilm-mediated infections by inducing the formation of yeast cells that are then easily dispersed from mature biofilms [15].

## Pheromones Stimulate Both Biofilm Formation and Sexual Reproduction

**Figure 2:**
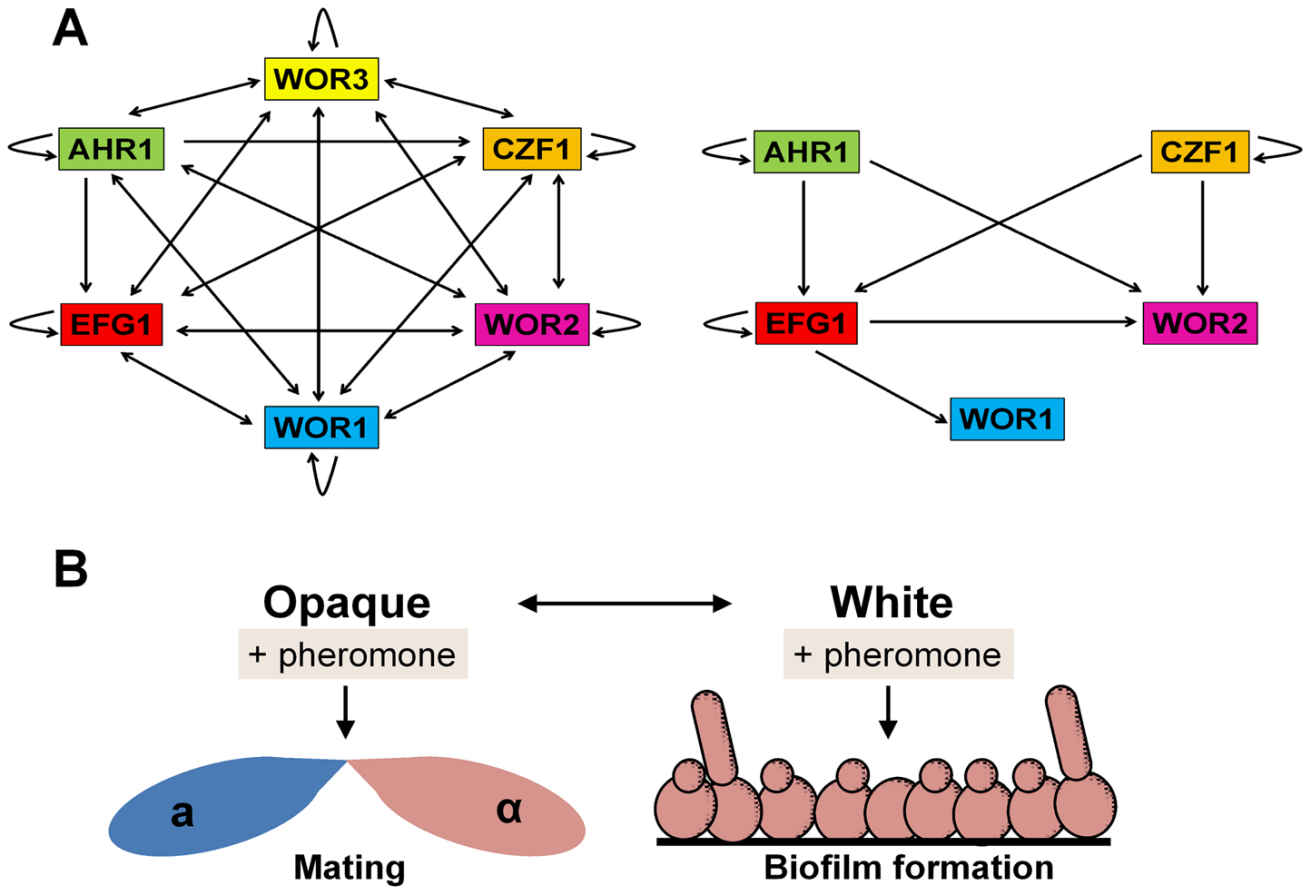
*C. albicans* white and opaque cells respond differently to pheromone. (**A**) The transcriptional network regulating formation of the opaque cell state (left) and white cell state (right) in *C. albicans*. Arrows indicate binding of a transcription factor to the promoter of another transcription factor, as determined by Hernday and coworkers [19]. (**B**) Schematic of the differential response of *C. albicans* white and opaque cells to pheromone. Pheromones induce mating responses in opaque cells but biofilm formation in white cells.

Long thought to be asexual, mating was discovered in *C. albicans* over a decade ago [16], [17]. In order to mate, *C. albicans* cells must be homozygous at the mating-type-like (*MTL*) locus and undergo a phenotypic switch from the white state to the mating-competent opaque state [18], [19]. The white-opaque switch is regulated by interacting transcriptional feedback loops and these lead to stable expression of Wor1, the master regulator of the opaque state [20] (**Figure 2A**). Following switching to opaque, **a** and α cells undergo mating ∼10^6^ times more efficiently than cells in the white state.

Notably, only opaque cells secrete sexual pheromones, yet both white and opaque **a** and α cells can respond to pheromones secreted by the opposite mating type. While opaque cells form conjugation tubes and undergo mating, white cells become more adhesive, forming pheromone-induced sexual biofilms [21], [22] (**Figure 2B**). Sexual biofilms promote the stabilization of pheromone gradients between opaque mating partners, allowing these cells to locate one another more efficiently and to undergo mating [23]. Interspecies pheromone signaling between different *Candida* species can also drive biofilm formation in white cells and sexual mating in opaque cells, indicating a surprising level of promiscuity in sexual signaling [21]. Mechanistically, pheromone signaling in both white and opaque cells occurs via the same conserved MAPK signaling pathway and Ste12/Cph1 transcription factor [24], [25]. It therefore remains to be seen how distinct phenotypic outputs are generated by the two phenotypic states, as well as the *in vivo* consequence of sexual biofilm formation.

QS also influences mating of *C. albicans*. Farnesol is produced by white cells growing aerobically but not by opaque cells, regardless of whether they are grown in aerobic or anaerobic environments [26]. Farnesol has been shown to kill opaque cells and decrease the mating efficiency under aerobic conditions, while not affecting white cells [26]. Aerobic production of farnesol may therefore restrict opaque cell formation and *C. albicans* mating to anaerobic sites in the body.

## Interkingdom Interactions between *C. albicans* and Bacteria

*C. albicans* exists in many niches in the human body including the skin, oral cavity, gastrointestinal (GI), and reproductive tracts. Therefore, it inevitably encounters and interacts with many other microbial species, and these interactions affect the survival, colonization, and pathogenesis of the organisms involved.

The gram-negative bacterium *Pseudomonas aeruginosa* is often co-isolated with *C. albicans* from patients with hospital-acquired infections, particularly those linked with colonization of medical devices such as catheters, patients with cystic fibrosis, and burn victims [27]. These two microbial species exhibit extensive crosstalk through secreted signaling molecules. *P. aeruginosa* harbors two QS systems and is able to establish an infection by attaching to and forming biofilms on *C. albicans* filaments, which, in turn, restricts their growth and causes death of the fungal cell [28]. Pyocyanin, haemolytic phospholipase C, phenazines, as well as other virulence factors, including GacA, LasR, RhlR, and RpoN, have been shown to limit the growth of *C. albicans*. Moreover, phenazines impair *C. albicans* biofilm formation and alter its metabolism thereby further decreasing virulence [29], [30] (**Figure 1A**). *P. aeruginosa* is also able to suppress the yeast-hyphal transition by producing the QS signaling molecule 3-oxo-C12 homoserine lactone (HSL) [28] (**Figure 1B**). Other bacteria also secrete substances that repress hyphal growth including two proteases regulated by the Fsr QS system in *Enterococcus faecalis*, cis-2-dodecenoic acid (BDSF) in *Burkholderia cenocepacia*, and diffusible signal factor (DSF) in *Xanthomonas campestris* [31]–[33] (**Figure 1A**). Increased *C. albicans* virulence is observed in the presence of *P. aeruginosa*, especially in the context of burn wounds. This is thought to be due to LasB (pseudolysin), a proteolytic enzyme produced by *P. aeruginosa* [34]. LasB has been implicated in playing a role in swarming motility and biofilm formation, and it is possible that through its proteolytic activity LasB is generating an amino acid signal that allows for increased biofilm formation and virulence.

Bacterial species that comprise the normal microbiota can also inhibit *C. albicans* from colonizing *in vivo* niches. For example, *Lactobacillus* sp., *Enterococcus faecalis*, and other bacterial flora restrict *C. albicans* colonization through the production of signaling molecules such as indole and metabolic by-products of lactic acid bacteria, which regulate factors responsible for the formation of filaments and biofilms [35]–[37]. Other proposed mechanisms by which commensal bacteria prevent *C. albicans* colonization include the production of hydrogen peroxide or organic acids, alteration of the host immune response, or by physically blocking bodily niches thereby preventing fungal adherence and invasion [37], [38]. Hence, it is not surprising that broad-spectrum antibiotic use is associated with *C. albicans* infections, and a treatment option for these infections includes the use of probiotics to repopulate the normal flora [37].

Bacteria also provide fungi with compounds that can enhance fungal virulence and, conversely, fungi can enhance bacterial virulence. For example, endotoxin (LPS) from *Escherichia coli* is considered an important contributor to virulence in co-infection experiments, and it has recently been shown that *C. albicans* responds directly to LPS [39], [40]. In addition, bacterial peptidoglycan molecules present in human serum induce hyphae formation in *C. albicans*, promoting tissue invasion and pathogenesis by this species [41]. *C. albicans* can also increase the virulence of bacterial pathogens such as *E. faecalis, Staphylococcus aureus*, and *Serratia marcescens*, as co-infection results in more severe disease than infection with the bacterial species alone [42]. Presumably, unidentified QS molecules and other virulence determinants are responsible for signaling between the different species thereby resulting in increased virulence.

Bacterial and fungal species are able to form mixed-species biofilms in oral environments, burn wounds, catheters, and other niches. These biofilms protect the microbial community from environmental pressures such as antibiotics and the host immune system. In the oral cavity, commensal *Streptococcus* species adhere to *C. albicans* cell wall proteins and adhesins including SspA, SspB, and Als3, thereby enhancing biofilm formation [43], [44]. *Streptococcus* species can also absorb protein components from saliva resulting in increased adherence and hyphal development in *C. albicans*, strengthening the biofilm and providing additional places for *Streptococcus* cells to bind [44]. Extracellular matrix production by *S. epidermidis* can inhibit penetration of antifungal drugs such as fluconazole in mixed-species biofilms [13].

Together, these findings reveal the complexities of mixed-species biofilms and the role that these structures play in responses to antimicrobial therapy. It is likely that these interactions represent the proverbial tip of the iceberg, and that further studies will be necessary to define how microbial species affect colonization and infection by *Candida* species, and for developing medical interventions that target these human pathogens.
